# Combinatory antibiotic therapy increases rate of bacterial kill but not final outcome in a novel mouse model of *Staphylococcus aureus* spinal implant infection

**DOI:** 10.1371/journal.pone.0173019

**Published:** 2017-02-28

**Authors:** Yan Hu, Vishal Hegde, Daniel Johansen, Amanda H. Loftin, Erik Dworsky, Stephen D. Zoller, Howard Y. Park, Christopher D. Hamad, George E. Nelson, Kevin P. Francis, Anthony Scaduto, Nicholas M. Bernthal

**Affiliations:** 1 Department of Orthopaedic Surgery, David Geffen School of Medicine at University of California Los Angeles, Santa Monica, California, United States of America; 2 Department of Medicine, Division of Infectious Diseases, Vanderbilt University Medical Center, Nashville, Tennessee, United States of America; Laurentian, CANADA

## Abstract

**Background:**

Management of spine implant infections (SII) are challenging. Explantation of infected spinal hardware can destabilize the spine, but retention can lead to cord compromise and biofilm formation, complicating management. While vancomycin monotherapy is commonly used, *in vitro* studies have shown reduced efficacy against biofilm compared to combination therapy with rifampin. Using an established *in vivo* mouse model of SII, we aim to evaluate whether combination therapy has increased efficacy compared to both vancomycin alone and infected controls.

**Methods:**

An L-shaped, Kirschner-wire was transfixed into the L4 spinous process of 12-week-old C57BL/6 mice, and inoculated with bioluminescent *Staphylococcus aureus*. Mice were randomized into a vancomycin group, a combination group with vancomycin plus rifampin, or a control group receiving saline. Treatment began on post-operative day (POD) 7 and continued through POD 14. *In vivo* imaging was performed to monitor bioluminescence for 35 days. Colony-forming units (CFUs) were cultured on POD 35.

**Results:**

Bioluminescence peaked around POD 7 for all groups. The combination group had a 10-fold decrease in signal by POD 10. The vancomycin and control groups reached similar levels on POD 17 and 21, respectively. On POD 25 the combination group dropped below baseline, but rebounded to the same level as the other groups, demonstrating a biofilm-associated infection by POD 35. Quantification of CFUs on POD 35 confirmed an ongoing infection in all three groups.

**Conclusions:**

Although both therapies were initially effective, they were not able to eliminate implant biofilm bacteria, resulting in a rebound infection after antibiotic cessation. This model shows, for the first time, why histologic-based, static assessments of antimicrobials can be misleading, and the importance of longitudinal tracking of infection. Future studies can use this model to test combinations of antibiotic therapies to see if they are more effective in eliminating biofilm prior to human trials.

## Introduction

Spinal implant infections (SII) are a disastrous complication of spine surgery, requiring lengthy hospitalizations, repeat surgeries, and protracted courses of intravenous and oral antibiotics. These infections often result in considerable morbidity for the patient, including neurological compromise, disability, and possibly death. In addition, economic costs are significant, with treatment of a single case averaging approximately $900,000.[1] Despite advances in aseptic surgical technique and perioperative antibiotic management, the rate of SII is still reported at 3–8% in elective spine surgery.[2–6] For multilevel or revision surgeries, rates of infection have been reported to be 65% higher than for primary cases.[7] As the demand for spine surgery continues to rise, so will the prevalence of these devastating infections, making their prevention and treatment critical.[8]

SII remains a unique challenge in orthopedics, as hardware removal is often not an option due to the potentially destabilizing effects of spinal hardware explantation prior to bony fusion, risking catastrophic surgical failure.[9] In addition, instrumentation itself results in 28% higher infection rates when compared to spinal surgery without the use of implants, as the avascular surface provides a nidus for microbial growth and eventual biofilm formation.[10] Once a biofilm layer is established on the implant surface, bacterial susceptibility to antibiotic and immune cell penetrance is reduced 1000-fold.[11] Since the current standard of treatment for SII relies on IV antibiotics +/- surgical debridement, the selection of antibiotic is critical.[9,12–15]

The optimal antibiotic regimen for biofilm treatment remains controversial. These infections typically arise from *Staphylococcus* species like *S*. *aureus*, the leading causative agent found in approximately 50% of cases of SII.[10] *S*. *epidermidis* is also a common cause and is associated with spinal implants. Given the established efficacy of the glycopeptide class against *Staphylococcus*, many surgeons choose to treat SII with IV vancomycin monotherapy.[9] However, *in vitro* studies have shown that vancomycin has reduced efficacy in the presence of biofilm, requiring concentrations of antibiotic that would be deemed toxic in humans.[16] Alternatively, the ability of rifampin to penetrate biofilm has been previously espoused as a rationale to utilize it against implant infections.[17–19] The Infectious Disease Society of America (IDSA) has recommended combination therapy with rifampin for acute staphylococcal SII.[20] This recommendation has been supported by *in vitro* studies, which show a synergistic effect when combination rifampin therapy is used in the setting of biofilm formation.[17–19,21–23] An optimized antibiotic therapy regimen for SII could lead to improved clearance of bacterial biofilm from implants, decreasing the need for repeated surgical debridement and thus reducing morbidity associated with SII.

An *in vivo* mouse model of SII in which bioluminescent *S*. *aureus* is inoculated onto a metallic orthopedic implant inserted into the L4 spinous process has recently been developed.[24] As there is a clinical impetus to improve the antibiotic treatment for SII and a lack of *in vivo* data, this model has the power to provide a rapid and cost effective platform through which to compare efficacies of antibiotics and combination therapies *in vivo* before transitioning to large-scale studies in human subjects. Thus, we used this established mouse model of SII to evaluate whether combination therapy with vancomycin plus rifampin has increased efficacy compared with vancomycin monotherapy in the treatment of a *S*. *aureus* SII with retention of the implant. We hypothesize that a combined therapy of rifampin and vancomycin will be synergistic in its treatment of bacterial biofilm and result in improved bacterial kill when compared to vancomycin alone.

## Materials and methods

### Ethics statement

All animals were handled in strict accordance with good animal practice as defined in the federal regulations as set forth in the Animal Welfare Act (AWA), the 1996 Guide for the Care and Use of Laboratory Animals, PHS Policy for the Humane Care and Use of Laboratory Animals, as well as our institution’s policies and procedures as set forth in the Animal Care and Use Training Manual, and all animal work was approved by the Chancellor’s Animal Research Committee (ARC#: 2012-104-11D).

### *Staphylococcus aureus* bioluminescent strain

The bioluminescent *S*. *aureus* strain Xen36 (PerkinElmer, Hopkinton, MA) was used in all experiments. The strain was derived from the parental strain ATCC 49525 (Wright), a clinical isolate obtained from a patient with *S*. *aureus* bacteremia.[25] Xen36 possesses a gram-positive optimized luxABCDE operon modified from the bacterial insect pathogen *Photorhabdus luminescens* in a stable bacterial plasmid that is maintained in all progeny. Live, actively metabolizing Xen36 bacteria emit a blue-green light with a maximal emission wavelength at approximately 490 nm.

### Preparation of *S. aureus* for inoculation of the spine implant

*S*. *aureus* Xen 36 possesses a kanamycin resistance marker on the *lux* operon. Bacteria were streaked onto tryptic soy agar plates (tryptic soy broth [TSB] plus 1.5% Bacto agar [BD Biosciences, Franklin Lakes, NJ]) containing 200ug/ml kanamycin (Sigma-Aldrich) and grown overnight at 37°C to select for Xen36 as previously described.[26] Single colonies of Xen36 were cultured in TSB and grown overnight at 37°C in a shaking incubator (240 rpm) (MaxQ 4450; Thermo, Waltham, MA). Mid-logarithmic phase bacteria were obtained after a 2-hour subculture of a 1:50 dilution of the overnight culture. Bacterial cells were pelleted, resuspended and washed 3 times in phosphate buffered saline (PBS). Bacterial inocula (1x10^3^ CFU/ml) were estimated by measuring the absorbance at 600 nm (Biomate 3; Thermo).

### Mice

Twelve-week old male C57BL/6 mice were used in all experiments.[27,28] Animals were kept at 3 mice per cage in standard cages with a 12-hr light and dark cycle. They were fed a standard pellet diet with free access to bottled water. Assessments were carried out daily by veterinary staff to ensure the well being of all animals throughout the experiment.

### Mouse surgical procedures

The institution’s Animal Research Committee approved all procedures. Mice were anesthetized via inhalation isoflurane (2%). The surgical procedure for this mouse model of spine implant infection was performed as previously described.[24] In brief, a custom, L-shaped, orthopaedic-grade 0.1 mm diameter stainless steel Kirschner-wire (Modern Grinding, Port Washington, WI) was press-fit into the L4 spinous process with the short arm of the implant in the spine and the long arm oriented longitudinally along the spine heading cranially. An inoculum of Xen36 (1x10^3^ CFUs) in 2μl PBS was pipetted onto the 90-degree bend of the implant. Deep 5–0 Vicryl sutures were used to close the muscle layer and a running 5–0 Vicryl was used to approximate the skin. For analgesia, non-sustained release buprenorphine (0.1mg/kg given in 100μl of volume) (Par Pharmaceuticals, Spring Valley, NY) was injected subcutaneously prior to surgery and every 12 hours postoperatively for 7–14 days as needed. A high-resolution AP radiograph (Faxitron LX-60 DC-12 imaging system) was taken immediately after surgery to ensure proper placement of the implant.

### Antibiotic therapy

Prior to surgery, mice were randomized to one of two treatment groups or a control group (n = 10 per group). Mice in the vancomycin group were subcutaneously administered a therapeutic dose of vancomycin (110 mg/kg twice daily) (Mylan, Cannonsburg, PA), which approximated the area under the curve (AUC) of 440 μg⋅h/ml for typical human exposure for vancomycin (1g twice daily).[29–31] Mice in the combination therapy group were administered a therapeutic subcutaneous mouse dose of rifampin (25 mg/kg daily) (Fresenius Kabi, Lake Zurich, IL) in addition to vancomycin therapy (110 mg/kg twice daily).[32] This rifampin dose was chosen based on previously published studies of various mouse models of staphylococcal infection.[32–36] All antibiotic therapy and sham injections of sterile saline were initiated on postoperative day 7 and continued through postoperative day 14 to mimic the treatment of an established SII. The MICs for Xen36 were ≤0.5 μg/ml for vancomycin and ≤0.5 μg/ml for rifampin.

### *In vivo* bacterial burden as measured by *in vivo* bioluminescence imaging

To obtain noninvasive measurement of bacterial burden, mice were anesthetized with inhalation isoflurane (2%), the dorsal region of the mouse was shaved, and *in vivo* bioluminescence imaging was performed using the Xenogen IVIS Lumina II (PerkinElmer, Hopkinton, MA) on post-operative days (POD) 0, 1, 3, 5, 7, 10, 14, 17, 21, 25, 28, 32, and 35 as previously described.[26,37–39] The Xen36 bioluminescent signals were obtained with a 5-minute acquisition time, a 13 cm field of view (FOV), and medium binning settings. Data are presented on a color scale overlaid on a grayscale photograph of mice and quantified as total flux (photons per second (s) per cm^2^ per steradian (sr) [photons/s/cm^2^/sr]) within a circular region of interest (1X10^3^ pixels) using Living Image software (PerkinElmer, Hopkinton, MA).

### Quantification of bacteria adhering to the implants and in the peri-implant tissue

To confirm that the *in vivo* bioluminescence signal accurately represented the bacterial burden *in vivo*, mice were euthanized on POD 35 using carbon dioxide exposure and secondary conformation by cervical dislocation. Traditional colony-forming unit (CFU) counts were then performed from bacteria adherent to the surgical implant and in the tissue surrounding the implant. Bacteria in the peri-implant tissue were isolated by homogenizing the surrounding soft tissue and infected bone of the spinous process (Pro200 Series homogenizer; Pro Scientific, Oxford, CT). Bacteria adherent to the implants were detached by sonication in 1 ml 0.3% Tween-80 in TSB for 10 min followed by vortexing for 5 minutes. Serial dilutions were plated and cultured overnight as previously described.[26] The number of bacterial CFUs obtained from the peri-implant tissue and the implants was determined by counting CFUs after overnight culture of plates.

### Statistical analysis

Each group had 10 mice based on previous studies from our group showing that 10 animals/group was necessary to attain statistical significance.[24,26,40] Data between two groups was compared using Student’s t-test (one or two-tailed where indicated), while data between three or more groups was compared using a one-way ANOVA. All data are expressed as mean ± standard error of the mean (SEM). Values of p< 0.05 were considered statistically significant.

## Results

### Efficacy of systemic antibiotic therapy on *in vivo* bioluminescent signals

**Fig 1:**
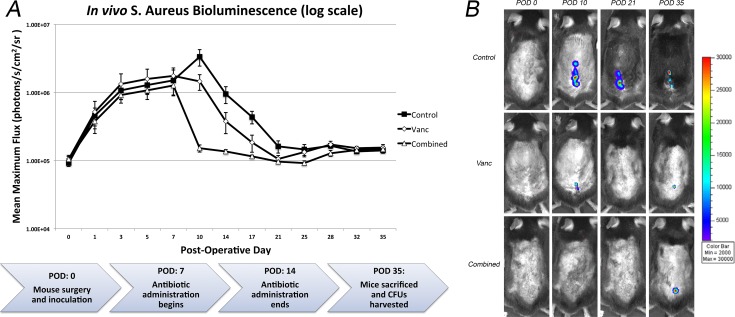
Measurement of bacterial burden using *in vivo* bioluminescence to POD 35. 1x10^3^ CFU of *S*. *aureus* possessing the bioluminescent construct in a stable plasmid (Xen36) were inoculated into the L4 spinous process of mice (n = 10 mice per group) in the presence of a stainless steel implant. (A) Bacterial counts as measured by in vivo *S*. *aureus* bioluminescence (mean maximum flux [photons/s/cm2/sr] ± sem [logarithmic scale]), with a flow diagram of the experimental protocol below. On POD 7, antibiotic administration began with vancomycin, a combination of vancomycin and rifampin or a sterile saline control. Antibiotic administration was stopped on POD 14. On POD 35, mice were sacrificed and CFUs from the implant and surrounding tissue were measured. (B) Representative in vivo *S*. *aureus* bioluminescence on a color scale overlaid on top of a grayscale image of mice.

Control mice treated with sterile saline had bioluminescent signals that peaked on POD 10 (3.35x10^6^ ± 9.14x10^5^ photons/s/cm^2^/sr) and remained above 1.00x10^5^ photons/s/cm^2^/sr throughout the duration of the experiment (35 days), modeling a chronic SII (Fig 1). A significant reduction in bioluminescent signal (approximately 2 fold) was observed with vancomycin monotherapy when compared to control mice from POD 10–21 (p<0.03). After this time point there was no difference in signal between the two groups. Combination therapy with vancomycin plus rifampin resulted in a rapid initial reduction in bioluminescent signal compared to controls on POD 10 (approximately 20 fold), which then remained lower until POD 28 (p<0.01), after which time there was no difference in signal between the combination and control groups. There was also a reduction in bioluminescent signal when comparing combination therapy to vancomycin monotherapy from POD 10–17 (approximately 2 fold) (p<0.05), after which there was no difference between the two groups. At final follow up (POD35), there were no differences in bacterial burden between any of the three groups.

### Quantification of CFUs from the implant and peri-implant bone and soft tissue

**Fig 2:**
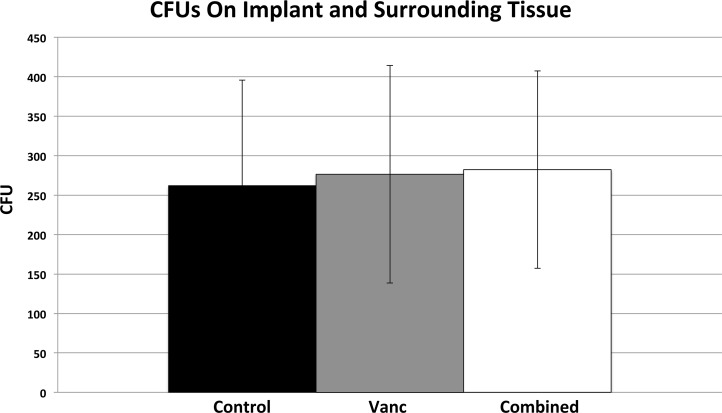
Confirmation of bacterial burden using CFU counts. At POD 35, mice were sacrificed, pins were sonicated, tissue was homogenized and bacteria were cultured and counted.

On POD 35, peri-implant bone and soft tissue as well as the implants themselves were harvested, and bacterial burden was quantified using CFU counts (Fig 2). There was no difference in the CFU counts between control, vancomycin monotherapy, and combination therapy mice (2.62x10^2^ ± 1.34x10^2^ CFUs, 2.76x10^2^ ± 1.38x10^2^ CFUs, and 2.82x10^2^ ± 1.25x10^2^ CFUs, respectively). This corroborates the bioluminescent data showing no difference between the three groups in terms of bioluminescent signal at POD 35.

## Discussion

The selection of antibiotic therapy for SII represents a significant challenge, as hardware typically must be retained to prevent potentially devastating destabilization of the spine. Biofilm has been shown to form as soon as 1 day after infection, with a robust biofilm present at 7 days.[41] Successfully treating biofilm infection necessitates the use of antibiotics that are effective against senescent bacteria. Although there is *in vitro* data indicating that combination therapy with rifampin is more effective against biofilm, there is a lack of *in vivo* data.[17–19,23] We thus used a previously established mouse model of SII to evaluate the efficacy of rifampin combination therapy when compared with vancomycin monotherapy in combating infection.[24]

The results of this study indicate that both vancomycin monotherapy and combination therapy with rifampin are not sufficient to eliminate a SII. Although combination therapy resulted in a considerably more rapid initial rate of kill compared to vancomycin alone, both treatments did result in a return to a baseline level of bioluminescent signal by POD 21. Yet after POD 21, and following the cessation of antibiotic therapy on POD 14, the signals for both groups increased back up to the level of control mice, indicating the persistence of a biofilm on the implant. Thus, while the rate of bactericidal activity was increased by combination therapy, neither regimen achieved eradication of the biofilm. This was corroborated by the *ex vivo* CFUs, which were present and not significantly different between the three groups at POD 35.

When interpreting this data, it is imperative to take into consideration the distinct pharmacokinetics of vancomycin and rifampin in humans versus mice, which can result in different levels of antibiotic exposure and efficacy. Firstly, serum protein binding differs between humans and mice for vancomycin (~35–50% versus ~25%) and rifampin (~97% versus ~90%), indicating that the level of active, unbound antibiotic is higher in mice than humans.[42,43] In addition, when humans are compared with mice, there is a longer half-life of vancomycin in humans (7.7h versus 2h), but a shorter half-life of rifampin (4h versus 12h).[44–47] This difference in half-life may result in a decreased rate of kill of vancomycin and increased rate of kill of rifampin therapy in this mouse model when compared to humans. These differences in the pharmacokinetics of the antibiotics highlight the difficulty of approximating human antibiotic exposures in mice. In future studies a range of antibiotic doses tested against a variety of *S*. *aureus* strains with varying antibiotic susceptibilities would ensure more robust pharmacokinetic and pharmacodynamic data that could more accurately evaluate the efficacy of these antibiotic therapies against SII in humans.

It is important to note that rifampin monotherapy is not used clinically due to a well documented rapid emergence of bacterial resistance, and was thus not tested in our mouse model.[48–50] While it could certainly be interesting to evaluate the curve dynamics for rifampin monotherapy, this data would not be clinically relevant and could give dangerous false-confidence in an otherwise well-negated drug regimen. Yet this study does confirm prior *in vitro* data indicating the utility of rifampin as an adjunctive agent.[23,51–53] Studies have shown that rifampin is able to penetrate biofilm, improve diffusion of a companion agent through biofilm, and reduce bacterial adherence to foreign body surfaces.[17–19,23] Differences in the mechanism of action of vancomycin and rifampin may also partially explain the increased efficacy of combination therapy. While vancomycin acts by disrupting cell wall synthesis, rifampin acts by inhibiting bacterial DNA-dependent RNA synthesis.[54,55] As bacteria in biofilms appear to have increased cell wall thickness, this may render vancomycin less effective when compared to its activity against planktonic bacteria, but may also improve rifampin penetrance into these thickened cell walls by interfering with their synthesis.[56] In spite of this, the synergy of these two bactericidal antibiotics was not sufficient to completely eliminate all of the bacteria in the biofilm, resulting in a return in the infection after antibiotic therapy had been stopped.

Looking at the curves for each of the three treatment groups in this experiment, the power of this longitudinal model of SII becomes apparent. Other infection models require sacrifice of animals and rely on CFU counts to measure bacterial burden, providing data at a single time point rather than tracking the course of the infection over time.[57–59] Taking this experiment as an example, if sacrifice and CFU counts were performed statically at POD 10, POD 21 or POD 35, completely different conclusions about the efficacy of vancomycin monotherapy versus combination therapy with rifampin would result (Fig 1). At POD 10, one would interpret that combination therapy is considerably more effective than vancomycin monotherapy. Interpreted at POD 21, it would appear that both treatment regimens were equally effective, and significant when compared to control animals. Finally, with counts taken at POD 35, it would appear that neither therapy was effective compared to control animals. Instead, by having bioluminescence as a surrogate for bacterial burden, the course of the infection can be tracked non-invasively and longitudinally without increased animal sacrifice, resulting in substantially more data and accuracy about the *in vivo* efficacy of these antibiotics in treating SII over time.

In conclusion, it is clear that both vancomycin monotherapy and combination therapy with rifampin are insufficient to clear a SII in this model. Even though it may appear that the infection has resolved, biofilm-associated bacteria remain on the implant. Thus, once antibiotics are discontinued, as was evidenced in this experiment, the infection persists at a quantifiable level. Using the power of a longitudinal model of SII, the progression of the infection can be traced, revealing more about the efficacy of antibiotic treatment over time than traditional infection models that are only capable of producing data at a single time point. In the future, this model can be used to examine different combinations of other antistaphylococcal drugs such as linezolid, tetracyclines (doxycycline, minocycline or tigecycline) or newer glycopeptides (daptomycin, telavancin, dalbavancin or oritavancin) to see if they perform better against SII than the currently accepted options of vancomycin monotherapy or combination therapy with rifampin. In addition, the course of antibiotic treatment could be studied longitudinally to help optimize the duration of suppressive therapy protocols. Expanding this model would result in a valuable preclinical system to screen different combinations of antibiotic therapy and duration before pursuing trials in humans.

## Supporting information

S1 File Bioluminescence and colony-forming unit data. (XLSX)

## References

[1] Abbey DM, Turner DM, Warson JS, Wirt TC, Scalley RD. Treatment of postoperative wound infections following spinal fusion with instrumentation. J Spinal Disord. 1995;8: 278–283. 8547767 10.1097/00002517-199508040-00003

[2] Verdrengh M, Thomas JA, Hultgren OH. IL-1 receptor-associated kinase 1 mediates protection against Staphylococcus aureus infection. Microbes and Infection. 2004;6: 1268–1272. 10.1016/j.micinf.2004.08.009 15555532

[3] Fang A, Hu SS, Endres N, Bradford DS. Risk factors for infection after spinal surgery. Spine. 2005;30: 1460–1465. 15959380 10.1097/01.brs.0000166532.58227.4f

[4] Levi AD, Dickman CA, Sonntag VK. Management of postoperative infections after spinal instrumentation. J Neurosurg. 1997;86: 975–980. 10.3171/jns.1997.86.6.0975 9171176

[5] Weinstein MA, McCabe JP, Cammisa FP. Postoperative spinal wound infection: a review of 2,391 consecutive index procedures. J Spinal Disord. 2000;13: 422–426. 11052352 10.1097/00002517-200010000-00009

[6] Picada R, Winter RB, Lonstein JE, Denis F, Pinto MR, Smith MD, et al Postoperative deep wound infection in adults after posterior lumbosacral spine fusion with instrumentation: incidence and management. J Spinal Disord. 2000;13: 42–45. 10710149 10.1097/00002517-200002000-00009

[7] Smith JS, Shaffrey CI, Sansur CA, Berven SH, Fu K-MG, Broadstone PA, et al Rates of infection after spine surgery based on 108,419 procedures: a report from the Scoliosis Research Society Morbidity and Mortality Committee. Spine. 2011;36: 556–563. 10.1097/BRS.0b013e3181eadd41 21192288

[8] Sivasubramaniam V, Patel HC, Ozdemir BA, Papadopoulos MC. Trends in hospital admissions and surgical procedures for degenerative lumbar spine disease in England: a 15-year time-series study. BMJ Open. 2015;5: e009011 10.1136/bmjopen-2015-009011 26671956 PMC4679892

[9] Hegde V, DS M, CK K, RC H. Management of postoperative spinal infections. World J Orthop. Baishideng Publishing Group Inc; 2012;3: 182–189. 10.5312/wjo.v3.i11.182 23330073 PMC3547112

[10] Chahoud J, Kanafani Z, Kanj SS. Surgical site infections following spine surgery: eliminating the controversies in the diagnosis. Front Med. 2014;1: 7. 10.3389/fmed.2014.00007 PMC4335387 25705620

[11] Olsen MA, Mayfield J, Lauryssen C, Polish LB, Jones M, Vest J, et al Risk factors for surgical site infection in spinal surgery. J Neurosurg. 2003;98: 149–155. 12650399

[12] Sierra-Hoffman M, Jinadatha C, Carpenter JL, Rahm M. Postoperative instrumented spine infections: a retrospective review. South Med J. 2010;103: 25–30. 10.1097/SMJ.0b013e3181c4e00b 19996837

[13] Pappou IP, Papadopoulos EC, Sama AA, Girardi FP, Cammisa FP. Postoperative infections in interbody fusion for degenerative spinal disease. Clinical Orthopaedics and Related Research. 2006;444: 120–128. 10.1097/01.blo.0000203446.06028.b5 16523136

[14] Gerometta A, Olaverri JCR, Bitan F. Infections in spinal instrumentation. Int Orthop. Springer-Verlag; 2012;36: 457–464. 10.1007/s00264-011-1426-0 22218913 PMC3282865

[15] Ahmed R, Greenlee JDW, Traynelis VC. Preservation of spinal instrumentation after development of postoperative bacterial infections in patients undergoing spinal arthrodesis. J Spinal Disord Tech. 2012;25: 299–302. 10.1097/BSD.0b013e31821fbf72 21617567

[16] Post V, Wahl P, Richards RG, Moriarty TF. Vancomycin displays time dependent eradication of mature Staphylococcus aureus biofilms. J Orthop Res. 2016. 10.1002/jor.23291 27175462

[17] Croes S, Beisser PS, Neef C, Bruggeman CA, Stobberingh EE. Unpredictable effects of rifampin as an adjunctive agent in elimination of rifampin-susceptible and -resistant Staphylococcus aureus strains grown in biofilms. Antimicrobial Agents and Chemotherapy. American Society for Microbiology; 2010;54: 3907–3912. 10.1128/AAC.01811-09 20606067 PMC2934976

[18] Dunne WM, Mason EO, Kaplan SL. Diffusion of rifampin and vancomycin through a Staphylococcus epidermidis biofilm. Antimicrobial Agents and Chemotherapy. 1993;37: 2522–2526. 8109913 10.1128/aac.37.12.2522 PMC192727

[19] Zheng Z, Stewart PS. Penetration of rifampin through Staphylococcus epidermidis biofilms. Antimicrobial Agents and Chemotherapy. 2002;46: 900–903. 10.1128/AAC.46.3.900-903.2002 11850284 PMC127480

[20] Liu C, Bayer A, Cosgrove SE, Daum RS. Clinical practice guidelines by the Infectious Diseases Society of America for the treatment of methicillin-resistant Staphylococcus aureus infections in adults and …. Clinical infectious …. 2011. 10.1093/cid/ciq146 21208910

[21] Rose WE, Poppens PT. Impact of biofilm on the in vitro activity of vancomycin alone and in combination with tigecycline and rifampicin against Staphylococcus aureus. J Antimicrob Chemother. 2009;63: 485–488. 10.1093/jac/dkn513 19109338

[22] Monzón M, Oteiza C, Leiva J, Amorena B. Synergy of different antibiotic combinations in biofilms of Staphylococcus epidermidis. J Antimicrob Chemother. 2001;48: 793–801. 11733463 10.1093/jac/48.6.793

[23] Saginur R, Stdenis M, Ferris W, Aaron SD, Chan F, Lee C, et al Multiple combination bactericidal testing of staphylococcal biofilms from implant-associated infections. Antimicrobial Agents and Chemotherapy. 2006;50: 55–61. 10.1128/AAC.50.1.55-61.2006 16377667 PMC1346774

[24] Dworsky E, Hegde V, Loftin A, Richman S, Hu Y, Lord E, et al A novel in vivo mouse model of implant related spine infection. J Orthop Res. 2016. 10.1002/jor.23273 PMC5268448 27116085

[25] Brand AM, de Kwaadsteniet M, Dicks LMT. The ability of nisin F to control Staphylococcus aureus infection in the peritoneal cavity, as studied in mice. Lett Appl Microbiol. 2010;51: 645–649. 10.1111/j.1472-765X.2010.02948.x 21029139

[26] Bernthal NM, Stavrakis AI, Billi F, Cho JS, Kremen TJ, Simon SI, et al A Mouse Model of Post-Arthroplasty Staphylococcus aureus Joint Infection to Evaluate In Vivo the Efficacy of Antimicrobial Implant Coatings. Planet PJ, editor. PLoS ONE. 2010;5: e12580–11. 10.1371/journal.pone.0012580 20830204 PMC2935351

[27] Kim M-H, Curry F-RE, Simon SI. Dynamics of neutrophil extravasation and vascular permeability are uncoupled during aseptic cutaneous wounding. Am J Physiol, Cell Physiol. 2009;296: C848–56. 10.1152/ajpcell.00520.2008 19176758 PMC2670654

[28] Kim M-H, Liu W, Borjesson DL, Curry F-RE, Miller LS, Cheung AL, et al Dynamics of neutrophil infiltration during cutaneous wound healing and infection using fluorescence imaging. J Invest Dermatol. 2008;128: 1812–1820. 10.1038/sj.jid.5701223 18185533 PMC2617712

[29] Hegde SS, Skinner R, Lewis SR, Krause KM, Blais J, Benton BM. Activity of telavancin against heterogeneous vancomycin-intermediate Staphylococcus aureus (hVISA) in vitro and in an in vivo mouse model of bacteraemia. J Antimicrob Chemother. 2010;65: 725–728. 10.1093/jac/dkq028 20139142

[30] Crandon JL, Kuti JL, Nicolau DP. Comparative efficacies of human simulated exposures of telavancin and vancomycin against methicillin-resistant Staphylococcus aureus with a range of vancomycin MICs in a murine pneumonia model. Antimicrobial Agents and Chemotherapy. 2010;54: 5115–5119. 10.1128/AAC.00062-10 20837760 PMC2981287

[31] Reyes N, Skinner R, Benton BM, Krause KM, Shelton J, Obedencio GP, et al Efficacy of telavancin in a murine model of bacteraemia induced by methicillin-resistant Staphylococcus aureus. J Antimicrob Chemother. 2006;58: 462–465. 10.1093/jac/dkl222 16735425

[32] Sakoulas G, Eliopoulos GM, Alder J, Eliopoulos CT. Efficacy of daptomycin in experimental endocarditis due to methicillin-resistant Staphylococcus aureus. Antimicrobial Agents and Chemotherapy. 2003;47: 1714–1718. 10.1128/AAC.47.5.1714-1718.2003 12709345 PMC153308

[33] van Ingen J, Aarnoutse RE, Donald PR, Diacon AH, Dawson R, Plemper van Balen G, et al Why Do We Use 600 mg of Rifampicin in Tuberculosis Treatment? Clin Infect Dis. 2011;52: e194–9. 10.1093/cid/cir184 21467012

[34] Mandell GL, Moorman DR. Treatment of experimental staphylococcal infections: effect of rifampin alone and in combination on development of rifampin resistance. Antimicrobial Agents and Chemotherapy. 1980;17: 658–662. 6901593 10.1128/aac.17.4.658 PMC283848

[35] Mandell GL, Vest TK. Killing of intraleukocytic Staphylococcus aureus by rifampin: in-vitro and in-vivo studies. J Infect Dis. 1972;125: 486–490. 5023643 10.1093/infdis/125.5.486

[36] Lobo MC, Mandell GL. Treatment of experimental staphylococcal infection with rifampin. Antimicrobial Agents and Chemotherapy. 1972;2: 195–200. 4790559 10.1128/aac.2.3.195 PMC444290

[37] Stavrakis AI, Niska JA, Shahbazian JH, Loftin AH, Ramos RI, Billi F, et al Combination Prophylactic Therapy with Rifampin Increases Efficacy against an Experimental Staphylococcus epidermidis Subcutaneous Implant-Related Infection. Antimicrobial Agents and Chemotherapy. 2014;58: 2377–2386. 10.1128/AAC.01943-13 24514089 PMC4023771

[38] Niska JA, Shahbazian JH, Ramos RI, Francis KP, Bernthal NM, Miller LS. Vancomycin-Rifampin Combination Therapy Has Enhanced Efficacy against an Experimental Staphylococcus aureus Prosthetic Joint Infection. Antimicrobial Agents and Chemotherapy. 2013;57: 5080–5086. 10.1128/AAC.00702-13 23917317 PMC3811477

[39] Pribaz JR, Bernthal NM, Billi F, Cho JS, Ramos RI, Guo Y, et al Mouse model of chronic post-arthroplasty infection: Noninvasive in vivo bioluminescence imaging to monitor bacterial burden for long-term study. J Orthop Res. 2011;30: 335–340. 10.1002/jor.21519 21837686 PMC3217109

[40] Bernthal NM, Pribaz JR, Stavrakis AI, Billi F, Cho JS, Ramos RI, et al Protective role of IL-1β against post-arthroplasty Staphylococcus aureus infection. J Orthop Res. 2011;29: 1621–1626. 10.1002/jor.21414 21445990 PMC3132302

[41] Traba C, Liang JF. Susceptibility of Staphylococcus aureus biofilms to reactive discharge gases. Biofouling. 2011;27: 763–772. 10.1080/08927014.2011.602188 21774615 PMC3181119

[42] Ackerman BH, Taylor EH, Olsen KM, Abdel-Malak W, Pappas AA. Vancomycin serum protein binding determination by ultrafiltration. Drug Intell Clin Pharm. 1988;22: 300–303. 3371190 10.1177/106002808802200404

[43] Knudsen JD, Fuursted K, Espersen F, Frimodt-Møller N. Activities of vancomycin and teicoplanin against penicillin-resistant pneumococci in vitro and in vivo and correlation to pharmacokinetic parameters in the mouse peritonitis model. Antimicrobial Agents and Chemotherapy. 1997;41: 1910–1915. 9303383 10.1128/aac.41.9.1910 PMC164034

[44] Reyes N, Skinner R, Kaniga K, Krause KM, Shelton J, Obedencio GP, et al Efficacy of telavancin (TD-6424), a rapidly bactericidal lipoglycopeptide with multiple mechanisms of action, in a murine model of pneumonia induced by methicillin-resistant Staphylococcus aureus. Antimicrobial Agents and Chemotherapy. 2005;49: 4344–4346. 10.1128/AAC.49.10.4344-4346.2005 16189117 PMC1251517

[45] Healy DP, Polk RE, Garson ML, Rock DT, Comstock TJ. Comparison of steady-state pharmacokinetics of two dosage regimens of vancomycin in normal volunteers. Antimicrobial Agents and Chemotherapy. 1987;31: 393–397. 3579256 10.1128/aac.31.3.393 PMC174739

[46] Forrest GN, Tamura K. Rifampin combination therapy for nonmycobacterial infections. Clin Microbiol Rev. 2010;23: 14–34. 10.1128/CMR.00034-09 20065324 PMC2806656

[47] Jayaram R, Gaonkar S, Kaur P, Suresh BL, Mahesh BN, Jayashree R, et al Pharmacokinetics-pharmacodynamics of rifampin in an aerosol infection model of tuberculosis. Antimicrobial Agents and Chemotherapy. 2003;47: 2118–2124. 10.1128/AAC.47.7.2118-2124.2003 12821456 PMC161844

[48] Zavasky DM, Sande MA. Reconsideration of rifampin: a unique drug for a unique infection. JAMA. 1998;279: 1575–1577. 9605905 10.1001/jama.279.19.1575

[49] Kadurugamuwa JL, Sin LV, Yu J, Francis KP, Purchio TF, Contag PR. Noninvasive Optical Imaging Method To Evaluate Postantibiotic Effects on Biofilm Infection In Vivo. Antimicrobial Agents and Chemotherapy. 2004;48: 2283–2287. 10.1128/AAC.48.6.2283-2287.2004 15155235 PMC415634

[50] Kadurugamuwa JL, Sin LV, Yu J, Francis KP, Kimura R, Purchio T, et al Rapid Direct Method for Monitoring Antibiotics in a Mouse Model of Bacterial Biofilm Infection. Antimicrobial Agents and Chemotherapy. 2003;47: 3130–3137. 10.1128/AAC.47.10.3130-3137.2003 14506020 PMC201124

[51] John A-K, Baldoni D, Haschke M, Rentsch K, Schaerli P, Zimmerli W, et al Efficacy of daptomycin in implant-associated infection due to methicillin-resistant Staphylococcus aureus: importance of combination with rifampin. Antimicrobial Agents and Chemotherapy. 2009;53: 2719–2724. 10.1128/AAC.00047-09 19364845 PMC2704655

[52] Raad I, Hanna H, Jiang Y, Dvorak T, Reitzel R, Chaiban G, et al Comparative activities of daptomycin, linezolid, and tigecycline against catheter-related methicillin-resistant Staphylococcus bacteremic isolates embedded in biofilm. Antimicrobial Agents and Chemotherapy. 2007;51: 1656–1660. 10.1128/AAC.00350-06 17353249 PMC1855569

[53] Parra-Ruiz J, Vidaillac C, Rose WE, Rybak MJ. Activities of high-dose daptomycin, vancomycin, and moxifloxacin alone or in combination with clarithromycin or rifampin in a novel in vitro model of Staphylococcus aureus biofilm. Antimicrobial Agents and Chemotherapy. 2010;54: 4329–4334. 10.1128/AAC.00455-10 20696880 PMC2944618

[54] Calvori C, Frontali L, Leoni L, Tecce G. Effect of rifamycin on protein synthesis. Nature. 1965;207: 417–418. 4957347 10.1038/207417a0

[55] Campbell EA, Korzheva N, Mustaev A, Murakami K, Nair S, Goldfarb A, et al Structural mechanism for rifampicin inhibition of bacterial rna polymerase. Cell. 2001;104: 901–912. 11290327 10.1016/s0092-8674(01)00286-0

[56] Reipert A, Ehlert K, Kast T, Bierbaum G. Morphological and genetic differences in two isogenic Staphylococcus aureus strains with decreased susceptibilities to vancomycin. Antimicrobial Agents and Chemotherapy. 2003;47: 568–576. 10.1128/AAC.47.2.568-576.2003 12543661 PMC151770

[57] Ofluoglu EA, Zileli M, Aydin D, Baris YS, Kuçukbasmaci O, Gonullu N, et al Implant-related infection model in rat spine. Arch Orthop Trauma Surg. Springer-Verlag; 2007;127: 391–396. 10.1007/s00402-007-0365-0 17522873

[58] Guiboux J-P, Ahlgren B, Patti JE, Bernhard M, Zervos M, Herkowitz HN. The Role of Prophylactic Antibiotics in Spinal Instrumentation: A Rabbit Model. Spine. 1998;23: 653 9549786 10.1097/00007632-199803150-00002

[59] Poelstra KA, Barekzi NA, Grainger DW, Gristina AG, Schuler TC. A Novel Spinal Implant Infection Model in Rabbits. Spine. 2000;25: 406 10707383 10.1097/00007632-200002150-00003

